# Two temporally validated diagnostic models for arterial stiffness using routine clinical indicators: a practical tool for resource-limited settings

**DOI:** 10.3389/fendo.2026.1891195

**Published:** 2026-07-16

**Authors:** Xiaohong Ma, Qiang Wang, Ting He, Lanqiqi Wu, Chao Shi

**Affiliations:** 1 People’s Hospital of Ningxia Hui Autonomous Region, Ningxia Medical University, Yinchuan, China; 2 Ningxia Institute of Clinical Medicine, People’s Hospital of Ningxia Hui Autonomous Region, Yinchuan, China; 3 School of Public Health, Ningxia Medical University, Yinchuan, China

**Keywords:** arterial stiffness, diagnostic model, easily accessible indicators, metabolic indices, temporal validation

## Abstract

**Background:**

Arterial stiffness is a strong independent predictor of cardiovascular diseases, yet its direct assessment (e.g., pulse wave velocity) is often impractical in resource-limited settings. The present study aimed to develop and validate diagnostic models for arterial stiffness using easily collectible clinical indicators.

**Methods:**

A cross-sectional analysis was conducted using data from two community-based cardiovascular health surveys in Yinchuan City, Ningxia, China. The derivation cohort (January 2020 to January 2021, n=2,440) and a temporally independent validation cohort (January 2025 to January 2026, n=2,512) were included. Two logistic regression models were developed: Model 1 included sex, age, waist circumference (WC), systolic blood pressure (SBP), and diastolic blood pressure (DBP); Model 2 additionally incorporated triglycerides (TG) and high-density lipoprotein cholesterol (HDL-C). Model performance was assessed using area under the receiver operating characteristic curve (AUC), calibration curves, bootstrap internal validation, and decision curve analysis (DCA). The models were compared against five metabolic indices: Lipid Accumulation Product (LAP), Weight−Adjusted Waist Index (WWI), Visceral Adiposity Index (VAI), triglyceride−glucose index (TyG), and TG/HDL−C ratio. Nomograms were constructed for clinical application.

**Results:**

In the derivation cohort, the AUCs for Model 1 and Model 2 were 0.926 and 0.927, respectively; in the validation cohort, both models achieved an AUC of 0.916. Both models significantly outperformed LAP, WWI, VAI, TyG, and TG/HDL−C (all *P* < 0.001). Calibration curves demonstrated good agreement between predicted and observed risks, and DCA confirmed positive net benefit across a wide range of threshold probabilities.

**Conclusions:**

The two models provide an accurate, easy-to-use tool for screening arterial stiffness, with temporal validation in an independent cohort from the same geographic region. Their excellent discriminative performance and favorable clinical utility make them particularly suitable for large-scale community-based screening or home-based self-management in resource-limited settings.

## 1 Introduction

Cardiovascular diseases (CVDs) remain the leading cause of death and disability worldwide, imposing a heavy burden on public health systems (1). Among well-established cardiovascular risk factors, arterial stiffness has been recognized as a strong and independent predictor, indicating early vascular damage (2, 3). Arterial stiffness increases pulse wave velocity and alters hemodynamics, thereby raising cardiac afterload and impairing target organ perfusion, significantly contributing to the development and progression of CVDs (3, 4). Therefore, early detection of arterial stiffness is crucial for CVD prevention.

However, assessment of arterial stiffness is often neglected in community-based cardiovascular risk screening programs, which mainly focus on traditional risk factors such as hypertension, diabetes mellitus, and dyslipidemia (5). Meanwhile, the gold-standard measurement of arterial stiffness, carotid-femoral pulse wave velocity (cf-PWV), relies on specialized, expensive equipment and trained personnel, limiting its large-scale application in resource-limited primary care settings (6, 7). Therefore, developing simple, economical, and accessible surrogate indicators for arterial stiffness has become an urgent priority for community-based cardiovascular risk stratification.

Insulin resistance (IR) is a key driver of arterial stiffness, and several simple metabolic indices have been developed as surrogate markers of IR. Among them, the triglyceride-glucose (TyG) index, triglyceride to high-density lipoprotein cholesterol (TG/HDL-C) ratio, lipid accumulation product (LAP), weight-adjusted waist circumference (WWI), and visceral adiposity index (VAI) have all been associated with arterial stiffness (8–13). However, these studies focused on associations rather than diagnostic performance. To date, no study has systematically evaluated the discriminative ability of these indices for identifying arterial stiffness. Moreover, whether a multivariable model integrating multiple easily obtainable indicators achieves superior diagnostic performance to any of these single indices (TyG, TG/HDL-C, LAP, WWI, or VAI) remains unknown.

To address these gaps, this study was designed to develop and validate diagnostic models for arterial stiffness using easily obtainable anthropometric and lipid indicators, and to systematically compare their performance against five established metabolic indices. A time−independent dual−cohort design was employed, with derivation (2020–2021) and validation (2025–2026) cohorts, based on data from two community−based cardiovascular health surveys in Yinchuan City, Ningxia, China. Two models were developed and compared against TyG, TG/HDL−C, LAP, WWI, and VAI. This approach enables effective community−based or home−based risk assessment for arterial stiffness, facilitating early CVD prevention in resource−limited regions.

## 2 Methods

### 2.1 Study population

This was a time-independent dual-cohort study based on the Ningxia Cardiovascular Disease Survey (NCDS), a repeated cross-sectional survey conducted every five years in Ningxia, China (14). Brachial-ankle pulse wave velocity (baPWV) measurements were only available for participants from Jinfeng District and Yongning County; therefore, the present study was restricted to these two sites.

Participants were eligible if they were permanent residents aged ≥18 years, provided informed consent, and completed all assessments. Exclusion criteria were: missing key data; outliers or extreme values; history of malignant tumor, end-stage renal disease, or severe liver disease; major surgery within 6 months; pregnancy; or severe mental disorders.

The derivation cohort included 2,440 participants from the 2020–2021 survey wave (January 1, 2020 to January 31, 2021). The temporally independent validation cohort included participants from the same two districts enrolled in the subsequent 2025–2026 survey wave (January 1, 2025 to January 31, 2026). Both waves used identical sampling strategies and measurement protocols, with no participant overlap.

### 2.2 Anthropometric and biochemical measurements

Participants reported to designated community health centers after an overnight fast of at least 8 hours. Anthropometric parameters, including waist circumference (WC), weight, and height, were measured using standardized procedures. After a 5-minute rest in a seated position, blood pressure was measured three times on the right arm using an electronic sphygmomanometer (OMRON, HBP-1120U), and the mean value was calculated for analysis.

Fasting venous blood samples (approximately 5 mL per participant) were collected into sodium heparin tubes and centrifuged at 1500 rpm for 10 minutes. The separated serum was aliquoted into cryovials, stored at -80 °C, and transported to the CIC Medical Laboratory Center in Beijing for analysis. Levels of low-density lipoprotein cholesterol (LDL-C), fasting plasma glucose (FPG), TG, HDL-C, and total cholesterol (TC) were measured using a Beckman Coulter AU5800 analyzer (USA) with Biosino reagents (Beijing). Glycated hemoglobin (HbA1c) was assayed using a Tosoh H-LC-723GX analyzer (Japan). All laboratory procedures adhered to standard quality control protocols.

### 2.3 Assessment of arterial stiffness

BaPWV was measured as an indicator of arterial stiffness using a Beijing M&B blood pressure and pulse wave velocity measurement device (MB3000). Before measurement, participants rested in a supine position for at least 10 minutes in a quiet, temperature-controlled room. With the participant in a supine position, cuffs were applied to both upper arms and ankles, and electrocardiogram and phonocardiogram sensors were attached (as per the device’s standard protocol). The device automatically measured and recorded baPWV values for both sides. Measurements were performed twice on each side, and the device automatically calculated the average baPWV value for the left and right sides, respectively. The higher value from the left or right side was used for analysis to represent the more severe degree of arterial stiffness. According to established consensus, arterial stiffness was defined as a baPWV value ≥ 1800 cm/s (15).

### 2.4 Calculated indices

TyG Index, LAP, WWI, and VAI were calculated using the following formulas:


TyG Index=ln[TG (mg/dL)×FPG (mg/dL)/2]

(16).


$$\begin{array}{l} LAP(female)=[WC(cm)-58]\times TG(mmol/L)\\ LAP\;(male)=[WC\;(cm)\;-\;65]\times TG\;(mmol/L) \end{array}$$

(17).


$$\text{WWI}=\frac{\text{WC}(\text{cm})}{\sqrt{\text{body weight }(\text{kg})}}$$

(18).


$$\begin{array}{l} VAI(female)=\frac{WC(cm)}{36.58+1.89\times BMI}\times \frac{TG(\text{mmol/L})}{0.90}\times \frac{1.12}{HDL-C(\text{mmol}/L)}\\ VAI(male)=\frac{WC(cm)}{39.68+1.88\times BMI}\times \frac{TG(\text{mmol/L})}{1.03}\times \frac{1.31}{HDL-C(\text{mmol}/L)} \end{array}$$

(19).

### 2.5 Statistical analysis

Descriptive statistics were used to describe participants’ anthropometric and laboratory measurements, with continuous variables presented as mean ± standard deviation (SD) for near-normal distributions and as median (interquartile range, IQR) for skewed distributions. Comparisons between the derivation cohort and the validation cohort were performed using Student’s t-test or Mann-Whitney U test for continuous variables and the chi-square test for categorical variables, as appropriate.

Two multivariable logistic regression models were constructed to diagnose arterial stiffness. Model 1 included age, gender, SBP, DBP, and WC, which are traditional cardiovascular risk factors readily available in community settings. Model 2 further incorporated TG and HDL-C based on Model 1, as dyslipidemia has been suggested to play an important role in arterial stiffness.

Discriminative performance of the models was evaluated using the area under the receiver operating characteristic curve (AUC) in both the derivation cohort and the validation cohort, and the AUCs of Model 1 and Model 2 were compared with those of traditional obesity-related indices (TyG, TG/HDL-C, LAP, WWI, and VAI) using the DeLong test. Model calibration was assessed using the Hosmer-Lemeshow (H-L) test and calibration curves to visually evaluate the agreement between predicted probabilities and observed outcomes. Bootstrap resampling (500 iterations) was performed for internal validation and to assess model stability. The optimal cutoff value was determined using the Youden index. Clinical utility was assessed using decision curve analysis (DCA) in both the derivation cohort and the validation cohort.

To address the calibration deviation observed in the external validation cohort, intercept recalibration was performed. Specifically, all regression coefficients of the model were kept unchanged, while the model intercept was re-estimated using the validation cohort data to align the mean predicted probability with the observed event rate in the validation cohort, thereby improving model calibration (20).

The nomograms were constructed based on the final multivariable logistic regression models. Points for each predictor were assigned by scaling the regression coefficients to a 0–100 point system, where the predictor with the largest absolute regression coefficient received the maximum points. The total points were then mapped to the corresponding predicted probability of arterial stiffness.

All statistical analyses were conducted using Stata software (version 18.0, StataCorp LLC, College Station, TX, USA), with a two−sided P < 0.05 considered statistically significant.

## 3 Results

### 3.1 Characteristics of the study population

**Table 1** displays the baseline characteristics of the study participants. A total of 4,952 participants were enrolled, including 2,440 (49.3%) in the derivation cohort and 2,512 (50.7%) in the validation cohort. Compared with the derivation cohort, the validation cohort showed significant differences in age (50 years vs. 45 years, *P* < 0.001) and most metabolic indicators, while no significant difference was observed in gender composition (52.62% female vs. 51.79% female, *P* = 0.558). Specifically, participants in the validation cohort were older and had significantly higher levels of BMI, WC, TG, TC, LDL-C, and FPG, and significantly lower levels of SBP, DBP, and HDL-C than those in the derivation cohort (all *P* < 0.01).The comparison of characteristics between arterial stiffness and Non-arterial stiffness in the training and validation cohorts is shown in **Supplementary Table 1**.

**Table 1:** Selected characteristics of participants in the derivation and validation cohorts.

Variables	Overall (N = 4952)	Derivation cohort (N = 2440)	Validation cohort (N = 2512)	P values A
Age (IQR)	47 (34,60)	45 (33,58)	50 (35,62)	<0.001 ^b^
Gender, n (%)				0.558 ^c^
Male	2367 (47.80)	1156 (47.38)	1211 (48.21)	
Female	2585 (52.20)	1284 (52.62)	1301 (51.79)	
Smoking, n (%)	1195 (24.13)	648 (26.56)	547 (21.78)	<0.001 ^c^
Drinking, n (%)	1174 (23.71)	720 (29.51)	454 (18.07)	<0.001 ^c^
Hypertension	1670 (33.72)	951 (38.98)	719 (28.62)	<0.001 ^c^
Diabetes	852 (17.04)	398 (16.31)	454 (18.07)	0.101 ^c^
SBP (mm Hg), mean ± SD	126.51 ± 19.34	130.00 ± 20.37	123.12 ± 17.65	<0.001 ^d^
DBP (mm Hg)	80.32 ± 10.84	81.08 ± 11.32	79.58 ± 10.29	<0.05 ^d^
BMI (kg/m2)	25.37 ± 3.91	25.21 ± 4.02	25.53 ± 3.81	<0.001 ^d^
WC (cm)	84.79 ± 11.44	84.04 ± 11.69	85.52 ± 11.15	<0.001 ^d^
TG (mmol/l)	1.74 ± 2.29	1.55 ± 1.50	1.92 ± 2.84	<0.001 ^d^
TC (mmol/l)	4.46 ± 1.01	4.25 ± 0.96	4.66 ± 1.02	<0.001 ^d^
LDL-C (mmol/l)	2.75 ± 0.82	2.60 ± 0.77	2.89 ± 0.85	<0.001 ^d^
HDL-C (mmol/l)	1.22 ± 0.31	1.24 ± 0.31	1.20 ± 0.32	<0.001 ^d^
FPG (mmol/l)	5.98 ± 1.59	5.85 ± 1.58	6.11 ± 1.59	<0.001 ^d^

IQR, interquartile range; SBP, systolic blood pressure; DBP, diastolic blood pressure; BMI, body mass index; WC, waist circumference; TG, triglyceride; TC, total cholesterol; LDL-C, low-density lipoprotein cholesterol; HDL-C, high-density lipoprotein cholesterol; FPG, fasting plasma glucose.

^a^P values were calculated to compare the characteristics of the derivation cohort and validation cohort.

^b^P values were obtained from the Mann-Whitney U test.

^c^P values were obtained from chi-square test.

^d^P values were obtained from the independent samples t-test.

### 3.2 Development of diagnostic models for arterial stiffness

**Table 2** presents the results of univariate and multivariate logistic regression analyses in the derivation cohort (n = 2,440). Univariate logistic regression analysis showed that male sex, age, WC, SBP, DBP, TG, and HDL-C were significantly associated with arterial stiffness (all *P* < 0.05). Based on these results, two multivariate models were constructed. Model 1 included sex, age, WC, SBP, and DBP. Model 2 further added TG and HDL-C to evaluate the incremental predictive value of lipid parameters. In multivariate analysis, age, WC, SBP, and DBP remained independent risk factors in both models (all *P* < 0.05), whereas sex lost statistical significance after adjustment for confounders, suggesting that the effect of sex is largely mediated by other metabolic and hemodynamic factors. In Model 2, elevated TG remained significantly associated with increased arterial stiffness risk (OR = 1.034, 95% CI: 1.000–1.071, *P* < 0.05), and lower HDL-C was also significantly associated with increased risk (OR = 0.688, 95% CI: 0.478–0.989, *P* < 0.05).

**Table 2:** Results of univariate and multivariate logistic regression analysis in the derivation cohort (n=2440).

Variables	Univariate analysis		Multivariate analysis (model 1) ^a^		Multivariate analysis (model 2) ^b^	
	Coefficients (95% CI) ^c^	OR (95% CI)	Coefficients (95% CI) ^c^	OR (95% CI)	Coefficients (95% CI) ^c^	OR (95% CI)
Gender						
Female	Reference	Reference	Reference	Reference	Reference	Reference
Male	0.185 (0.008, 0.362)	1.203 (1.008, 1.437)	-0.047 (-0.275,0.181)	0.954 (0.759, 1.199)	-0.084 (-0.316, 0.149)	0.920 (0.729, 1.160)
Age	0.126(0.116, 0.136)	1.134 (1.123, 1.145)	0.129 (0.117, 0.141)	1.138 (1.124, 1.152)	0.130 (0.118, 0.143)	1.139 (1.125, 1.154)
WC	0.057 (0.048, 0.065)	1.058 (1.050, 1.067)	0.022 (0.010, 0.033)	1.022 (1.010, 1.034)	0.020 (0.008, 0.032)	1.020 (1.008, 1.032)
SBP	0.053 (0.048, 0.058)	1.054 (1.049, 1.059)	0.022 (0.014, 0.029)	1.022 (1.014, 1.030)	0.022 (0.015, 0.029)	1.022 (1.015, 1.030)
DBP	0.033 (0.025, 0.041)	1.034 (1.026, 1.042)	0.033 (0.019, 0.047)	1.034 (1.019, 1.048)	0.033 (0.019, 0.047)	1.034 (1.019, 1.049)
TG	0.016 (-0.013, 0.044)	1.016 (0.987, 1.045)	–	–	0.034 (0.000, 0.068)	1.034 (1.000, 1.071)
HDL-C	-0.391 (-0.683, -0.100)	0.676 (0.505, 0.905)	–	–	-0.375 (-0.739, -0.011)	0.688 (0.478, 0.989)

SBP, systolic blood pressure; DBP, diastolic blood pressure; WC, waist circumference; TG, triglyceride; HDL-C, high-density lipoprotein cholesterol.

^a^Model 1 incorporated gender, age, WC, SBP and DBP, with an intercept of -18.823.

^b^Model 2 incorporated gender, age, WC, SBP, DBP, TG and HDL-C, with an intercept of -18.937.

^c^Coefficients represent log-transformed odds ratios. Univariate analysis was performed for each variable individually, and multivariate analysis was adjusted for all variables included in the respective model.

**Supplementary Table 2** shows that Model 1 has a sensitivity of 90.45% and specificity of 80.83%, with an optimal cutoff value of 0.101. Model 2 has a sensitivity of 91.36% and specificity of 82.88%, with an optimal cutoff value of 0.093. Both models yielded identical Youden indices (0.74), and the difference in optimal cutoffs reflects the different predicted probability distributions generated by the two models. The improvement in both sensitivity and specificity in Model 2 indicates that adding TG and HDL-C enhances the model’s ability to correctly identify individuals with and without arterial stiffness. Both models demonstrated strong predictive capabilities, with Model 2 performing slightly better.

### 3.3 Assessment of the diagnostic performance of the models

**Figure 1:**
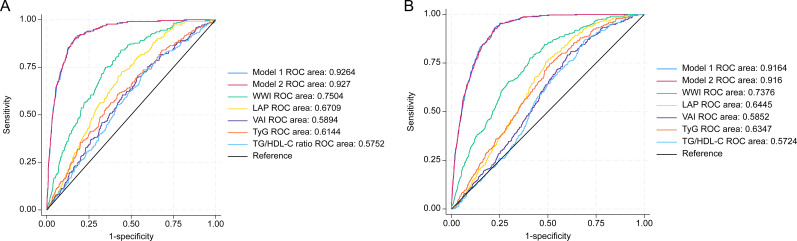
ROC curves of WWI, LAP, AI, TyG, TG/HDL-C and the models to identify the presence of arterial stiffness. **(A)** ROC curves of Model 1, Model 2, WWI, LAP, VAI, TyG, and TG/HDL-C for identifying the presence of arterial stiffness in the derivation cohort. **(B)** ROC curves of Model 1, Model 2, WWI, LAP, VAI, TyG, and TG/HDL-C for identifying the presence of arterial stiffness in the validation cohort. Abbreviations: WWI, Weight−Adjusted Waist Index; LAP, lipid accumulation product; VAI, visceral adiposity index; TyG, triglyceride-glucose index; TG/HDL-C, triglyceride to high-density lipoprotein cholesterol ratio.

In the derivation cohort, both Model 1 and Model 2 demonstrated excellent diagnostic performance for arterial stiffness, with AUCs of 0.926 and 0.927, respectively, significantly outperforming WWI (0.750), LAP (0.671), VAI (0.589), TyG (0.614), and TG/HDL-C (0.575) (all *P* < 0.001) (**Figure 1A**). These findings were confirmed in the validation cohort, where both Model 1 and Model 2 achieved AUCs of 0.916, respectively, again substantially exceeding the comparators: WWI (0.738), LAP (0.645), VAI (0.585), TyG (0.635), and TG/HDL-C (0.572) (all *P* < 0.001) (**Figure 1B**).

### 3.4 Model calibration and stability assessment

**Figure 2:**
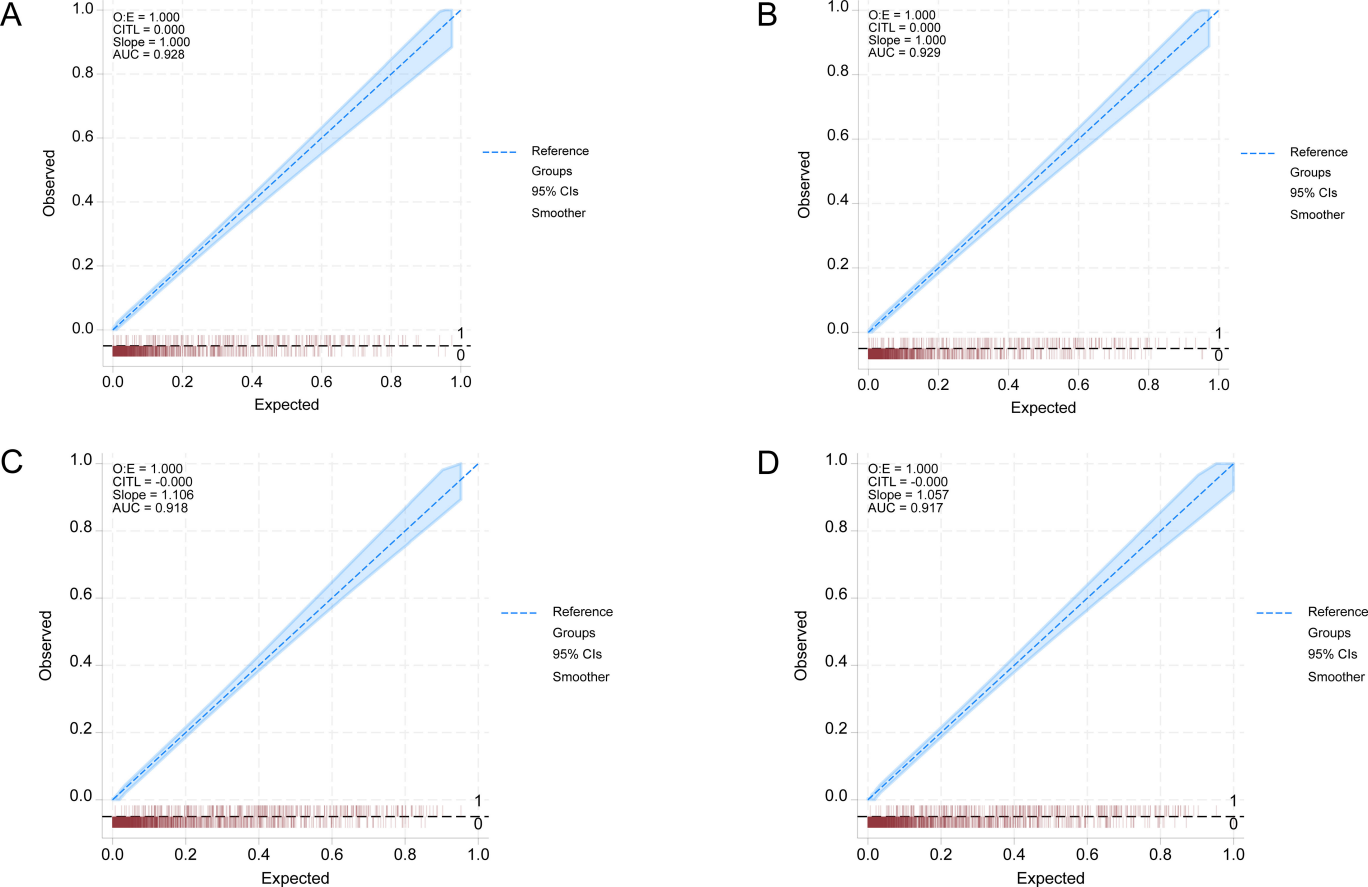
Calibration curves of Model 1 and Model 2 in the derivation and validation cohorts. **(A, B)** The Calibration curve for model 1 **(A)** and model 2 **(B)** to identify the presence of arterial stiffness in derivation cohort. **(C, D)** The Calibration curve for model 1 **(C)** and model 2 **(D)** to identify the presence of arterial stiffness in validation cohort.

Calibration curves were used to assess the consistency between the predicted probabilities of the models and the actual observed probabilities. As shown in **Figure 2**, in the derivation cohort, the calibration curves (smoother lines) of both Model 1 (**Figure 2A**) and Model 2 (**Figure 2B**) were highly consistent with the ideal reference line (45° dashed line), with observed probabilities closely matching predicted probabilities across the entire risk range, indicating excellent calibration. in the validation cohort, following intercept recalibration, the calibration curves of Model 1 (**Figure 2C**) and Model 2 (**Figure 2D**) also demonstrated good agreement with the ideal reference line. The predicted probabilities showed strong consistency with the observed probabilities across the entire risk range, with no systematic overestimation or underestimation, indicating that the models maintained good calibration in the validation cohort after recalibration. Hosmer-Lemeshow test confirmed good calibration for both models in both cohorts (all *P* > 0.05).

Bootstrap resampling with 500 iterations was performed to further assess model stability and overfitting (**Supplementary Figure 1**). In the derivation cohort, the calibration curves after resampling demonstrated that the predicted risk of arterial stiffness was highly consistent with the actual observed risk for both Model 1 (**Supplementary Figure 1A**) and Model 2 (**Supplementary Figure 1B**). The calibration points (groups) were closely distributed around the ideal reference line, and the smoother line showed minimal deviation across the entire risk range. The narrow gap between the predicted and observed risk curves confirmed the strong calibration of the models and ruled out significant overfitting.

### 3.5 Clinical utility assessment for the models

**Figure 3:**
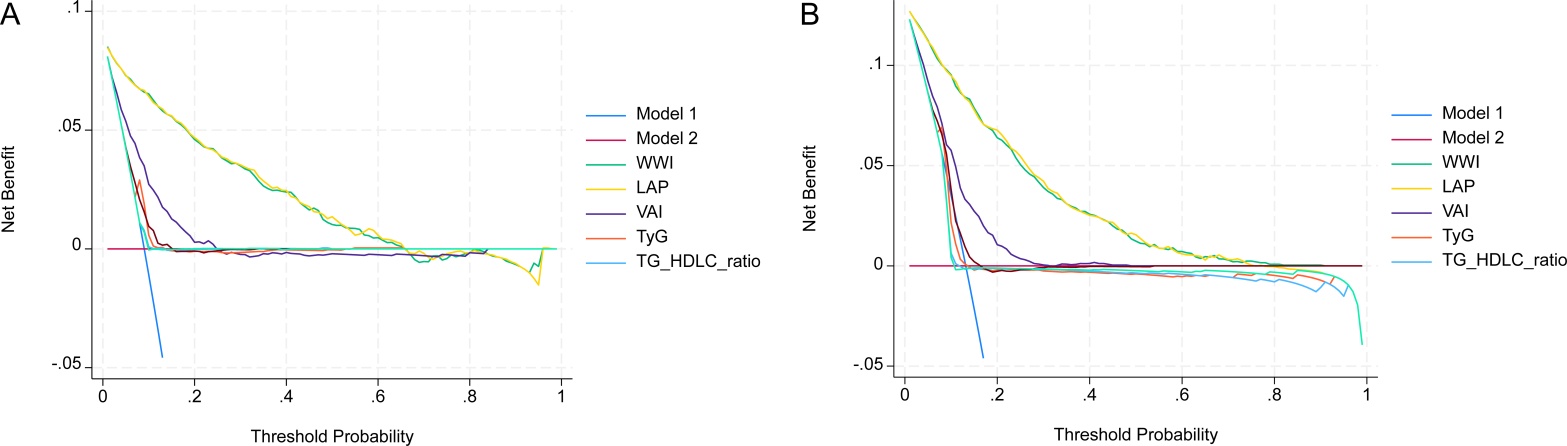
Decision curve analysis (DCA) for Model 1 and Model 2 in derivation and validation cohorts. **(A, B)** Decision curve analysis for Model 1, Model 2, WWI, LAP, VAI, TyG, and TG/HDL-C to identify the presence of arterial stiffness in the derivation **(A)** and validation **(B)** sets.

The clinical utility of both models was further confirmed by Decision Curve Analysis (**Figure 3**), which demonstrated substantial net benefit across a wide range of probability thresholds in both derivation and validation cohorts. These findings collectively support the practical value of both models for clinical risk stratification and assessment of arterial stiffness.

### 3.6 Nomogram construction for the models

**Figure 4:**
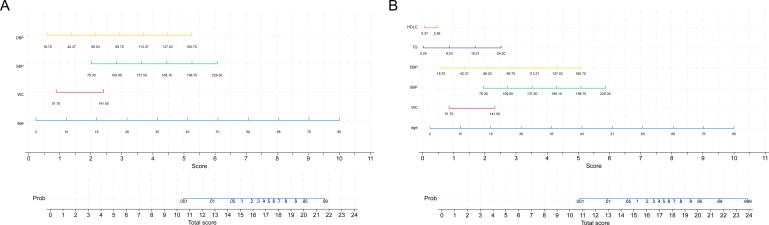
Nomogram model 1 **(A)** and model 2 **(B)** for arterial stiffness. Each indicator in the nomogram prediction model can get its corresponding score in the integral line in the middle of the graph, and then each corresponding score can be summed up one by one to obtain the total score, which corresponds to the probability of the occurrence of arterial stiffness. Points for each predictor were derived from the scaled regression coefficients of the final multivariable logistic regression model.

To facilitate practical clinical application, we developed a nomogram based on the risk prediction model (**Figure 4**). The nomogram demonstrates the contribution weight of each indicator to the risk score. Variables with higher point values play a more important role in risk prediction. The nomogram provides a one-step conversion from risk factor quantification to predicted risk probability, offering a simple, practical, and intuitive tool for rapid assessment of arterial stiffness risk in clinical practice.

## 4 Discussion

In this time-independent dual-cohort study of 4,952 participants from community-based cardiovascular health surveys, two diagnostic models for arterial stiffness were developed and temporally validated using easily obtainable clinical indicators. Both models demonstrated excellent discriminative ability, with AUCs of 0.926–0.927 in the derivation cohort and 0.916 for both models in the validation cohort, significantly outperforming five commonly used metabolic indices (TyG, TG/HDL-C, LAP, WWI, and VAI). Moreover, both models showed good calibration, stability against overfitting, and favorable clinical utility across a wide range of threshold probabilities. These findings suggest that the proposed models offer a practical and accessible approach for arterial stiffness risk assessment, particularly in resource-limited primary care settings where direct measurement of pulse wave velocity is not feasible.

Several simple metabolic indices (TyG, TG/HDL-C, LAP, WWI, and VAI) have been reported to be positively correlated with arterial stiffness in various populations. However, previous studies primarily focused on associations rather than systematically evaluating diagnostic performance. The present study extends this literature by directly comparing the discriminative ability of these indices and, more importantly, demonstrating that multivariable models incorporating multiple clinical parameters substantially outperform any single index. In the derivation cohort, the AUCs of individual indices ranged from 0.575 to 0.750, indicating only modest to moderate diagnostic performance. Among these, WWI performed relatively better (AUC = 0.750 in the derivation cohort and 0.738 in the validation cohort), which is consistent with recent studies highlighting the importance of body composition and central obesity in vascular health (21, 22). Nevertheless, the diagnostic accuracy of even the best-performing single index remained far below that of the multivariable models (AUC > 0.916). This substantial improvement likely reflects the multifactorial nature of arterial stiffness, which is influenced by hemodynamic, anthropometric, and lipid factors that are not fully captured by any single metabolic index. Previous studies have also shown that risk models combining multiple clinical indicators are generally superior to single biomarkers in predicting vascular diseases (23, 24).

Multivariate analysis revealed that age, WC, SBP, and DBP were independent risk factors for arterial stiffness in both models, consistent with extensive prior evidence (25, 26). Notably, sex was significantly associated with arterial stiffness in univariate analysis but lost significance after adjustment for confounders, suggesting that the observed sex difference is largely mediated by metabolic and hemodynamic factors rather than an independent biological effect. This finding aligns with previous studies reporting that adjustment for body composition or blood pressure attenuates sex-related differences in vascular stiffness (27). Model 2 incorporated TG and HDL-C in addition to the variables in Model 1. Both lipid parameters remained independently associated with arterial stiffness after multivariable adjustment, albeit with modest effect sizes (TG: OR = 1.034;HDL-C: OR = 0.813). The small magnitude of these ORs is not surprising, as arterial stiffness is a complex trait, and the incremental contribution of any single lipid parameter may be modest when strong predictors like age and blood pressure are already in the model. Nevertheless, the addition of TG and HDL-C improved model sensitivity (from 90.45% to 91.36%) and specificity (from 80.83% to 82.88%), indicating a modest but consistent enhancement in discriminative ability. This incremental improvement supports the inclusion of lipid parameters in risk models, particularly given the low cost and wide availability of routine lipid profiling in community settings. Although the AUC gain was modest, which is not unexpected given that Model 1 already achieved excellent performance, the consistent gains in sensitivity and specificity across both cohorts suggest that the addition of lipids provides meaningful refinement in individual risk stratification. In fact, accumulating evidence indicates that lipid parameters provide independent predictive value even beyond traditional risk factors (28–30).

Beyond discrimination, a well-performing diagnostic model must also demonstrate good calibration, defined as agreement between predicted and observed risks, to be clinically useful (31). The models showed excellent calibration in the derivation cohort, with calibration curves closely following the ideal 45° reference line. in the external validation cohort, intercept recalibration was performed, a common approach when predicted versus observed risks show systematic deviation across cohorts due to differences in baseline risk (32). After recalibration, both models maintained good calibration without systematic overestimation or underestimation. Bootstrap resampling with 500 iterations confirmed model stability and ruled out significant overfitting, as evidenced by the narrow gap between predicted and observed risk curves after resampling. These results support the robustness of the models for future external applications.

Having confirmed satisfactory discrimination, calibration, and stability, the clinical utility of the models was further evaluated using DCA. DCA demonstrated that both models provided positive net benefit across a wide range of threshold probabilities in both the training and validation cohorts. DCA is increasingly recognized as the preferred method for evaluating clinical utility because it incorporates the trade-off between true positives and false positives across clinically relevant decision thresholds (33). The DCA results indicate that using these models to guide clinical decisions, such as prioritizing patients for further vascular testing or lifestyle interventions, would yield net benefit over either “treat all” or “treat none” strategies acrosmost risk thresholds (34).

To facilitate direct clinical translation, nomograms were developed based on both models. These nomograms allow clinicians to compute an individual’s predicted probability of arterial stiffness by summing points assigned to each risk factor, without requiring specialized software. Notably, age and SBP contributed the most points in both nomograms, reflecting their dominant roles in the multivariate analysis (**Table 2**). This graphical tool is particularly valuable in primary care or low-resource settings where electronic risk calculators are unavailable. As an intuitive risk visualization tool, nomograms have been widely used for individualized risk prediction in cardiovascular diseases (35, 36).

Compared with three recently published diagnostic models for arterial stiffness, our models demonstrated superior predictive performance (AUC 0.926–0.927 vs. 0.795–0.877) while requiring fewer variables (five to seven routinely available indicators vs. six to seven variables, some requiring CT or specialized biomarkers) (37–39). Notably, our models were validated in a large temporally independent cohort (n=2,512), providing stronger evidence for generalizability than previous studies, which either lacked external validation or used smaller validation samples (n=399) (39).

Strengths of this study include the use of a time-independent external validation cohort, which provides a more rigorous test of model generalizability compared to internal validation methods such as cross-validation or bootstrap alone. The large sample size (n = 4,952) and the inclusion of participants from two distinct survey waves (2020–2021 and 2025–2026) reduce the likelihood of temporal or seasonal bias. Additionally, the models were systematically compared against five established metabolic indices using standardized performance metrics (AUC, calibration, DCA), facilitating direct comparison with future studies.

Several limitations should be acknowledged. First, both cohorts were derived from two districts in Yinchuan City, Ningxia, China. Although temporal separation was used to provide independent validation, it should be acknowledged that this represents time−dependent validation rather than validation across geographically or ethnically distinct populations. Therefore, the generalizability of our models to other populations, ethnicities, and healthcare settings remains uncertain, and external validation in more diverse cohorts is needed to confirm broader applicability. Second, the models were developed for diagnosis of existing arterial stiffness (baPWV ≥ 1800 cm/s) rather than prediction of future cardiovascular events. Longitudinal studies are needed to evaluate whether these models predict incident arterial stiffness or downstream CVD outcomes. Third, despite adjusting for major confounders, the potential influence of unmeasured factors such as physical activity, diet, medication use, and duration of metabolic diseases cannot be excluded.

## 5 Conclusion

In conclusion, two simple diagnostic models for arterial stiffness were developed and externally validated using easily obtainable clinical indicators. Both models demonstrated excellent discrimination (AUC > 0.916), good calibration, minimal overfitting, and favorable clinical utility, substantially outperforming five single metabolic indices. The accompanying nomograms provide practical tools for community-based risk assessment. These findings support the use of the models for early identification of individuals at high risk of arterial stiffness in resource-limited primary care settings, facilitating timely intervention for cardiovascular disease prevention.

## Data Availability

The raw data supporting the conclusions of this article will be made available by the authors, without undue reservation.
