# Supplementary material for: Two temporally validated diagnostic models for arterial stiffness using routine clinical indicators: a practical tool for resource-limited settings
Source: Front Endocrinol (Lausanne). 2026 Jul 16;17:1891195. doi: 10.3389/fendo.2026.1891195 (PMC13421444; doi:10.3389/fendo.2026.1891195)
Supplement: Supplementary file 1 [file DataSheet1.pdf]

Supplementary Table 1. Comparison of selected characteristics between Arterial stiffness and Non-Arterial stiffness in derivation and validation cohorts

Variables	Derivation cohort (n=2,440)				Validation cohort (n=2,512)			
	Overall (N=2440)	Arterial stiffness (N=220)	Non-arterial stiffness (N = 2220)	*P* values	Overall (N=2,512)	Arterial stiffness (N=331)	Non-arterial stiffness (N =2181)	*P* values
Age (IQR)	45 (33,58)	72 (65,77)	43 (33,55)	＜0.001 [a]	50 (35,62)	72 (63,76)	46 (33,57)	＜0.001 [a]
Gender, n (%)				0.051 [b]				0.320 [b]
Male	1156 (47.38)	118 (10.21)	1038 (89.79)		1211 (48.21)	168 (50.76)	1043 (47,82)	
Female	1284 (52.62)	102 (7.94)	1182 (92.06)		1301 (51.79)	163 (49.24)	1138 (52.18)	
Smoking, n (%)	648 (26.56)	58 (26.36)	590 (26.58)	0.946 [b]	547 (21.78)	55 (16.62)	492 (22.56)	＜0.05 [b]
Drinking, n (%)	720 (29.51)	47 (21.36)	673 (30.32)	＜0.05 [b]	454 (18.07)	44 (13.29)	410 (18.80)	＜0.05 [b]
Hypertension	951 (38.98)	199 (90.45)	752 (33.87)	＜0.001 [b]	719 (28.62)	221 (66.77)	498 (22.83)	＜0.001 [b]
Diabetes	398 (16.31)	81 (36.82)	317 (14.28)	＜0.001 [b]	454 (18.07)	44 (13.29)	410 (18.80)	＜0.05 [b]
SBP (mm Hg), mean±SD	130.00±20.37	154.65±19.62	127.55±18.76	＜0.001 [c]	123.12±17.65	139.42±17.24	120.65±16.35	＜0.001 [c]
DBP (mm Hg)	81.08±11.32	86.96±12.15	80.50±11.07	＜0.001 [c]	79.58±10.29	81.94±10.52	79.22±10.21	＜0.01 [c]
BMI (kg/m^2)	25.21±4.02	26.69±3.42	25.06±4.04	＜0.001 [c]	25.53±3.81	26.85±3.28	25.33±3.84	＜0.001 [c]
WC (cm)	84.04±11.69	91.11±9.73	83.34±11.64	0.063 [c]	85.52±11.15	91.22±8.69	84.65±11.23	0.989 [c]
TG (mmol/l)	1.55±1.50	1.73±1.68	1.53±1.48	＜0.05 [c]	1.92±2.84	1.91±0.96	1.92±3.03	0.149 [c]
TC (mmol/l)	4.25±0.96	4.45±0.92	4.23±0.96	＜0.05 [c]	4.66±1.02	4.74±1.20	4.65±0.99	＜0.05 [c]
LDL-C (mmol/l)	2.60±0.77	2.74±0.78	2.58±0.77	＜0.05 [c]	2.89±0.85	2.98±0.98	2.88±0.82	＜0.05 [c]
HDL-C (mmol/l)	1.24±0.31	1.22±0.34	1.24±0.31	0.401 [c]	1.20±0.32	1.16±0.27	1.21±0.32	＜0.001 [c]
FPG (mmol/l)	5.85±1.58	6.67±2.58	5.77±1.42	＜0.001 [c]	6.11±1.59	6.91±1.95	5.98±1.49	＜0.001 [c]

Abbreviations: SBP, systolic blood pressure; DBP, diastolic blood pressure; BMI, body mass index; WC, waist circumference; TG, triglyceride; TC, total cholesterol; LDL-C, low-density lipoprotein cholesterol; HDL-C, high-density lipoprotein cholesterol; FPG, fasting plasma glucose.

[a] *P* values for age were obtained from the Mann-Whitney U test.

[b] *P* values for categorical variables were obtained from the chi-square test.

[c] *P* values for other continuous variables were obtained from the independent samples t-test

Supplementary Table 2. Optimal cutoff values for diagnosing arterial stiffness of the models

Variable	Model 1 [a]	Model 2 [b]
Optimal cutoff	0.101	0.093
Sensitivity (%)	90.45	91.36
Specificity (%)	80.83	82.88
Youden index	0.74	0.74

Abbreviations: SBP, systolic blood pressure; DBP, diastolic blood pressure; WC, waist circumference; TG, triglyceride; HDL-C, high-density lipoprotein cholesterol.

[a] The model 1 incorporated gender, age, WC, SBP, and DBP.

[b] The model 2 incorporated gender, age, WC, SBP, DBP, TG and HDL-C.

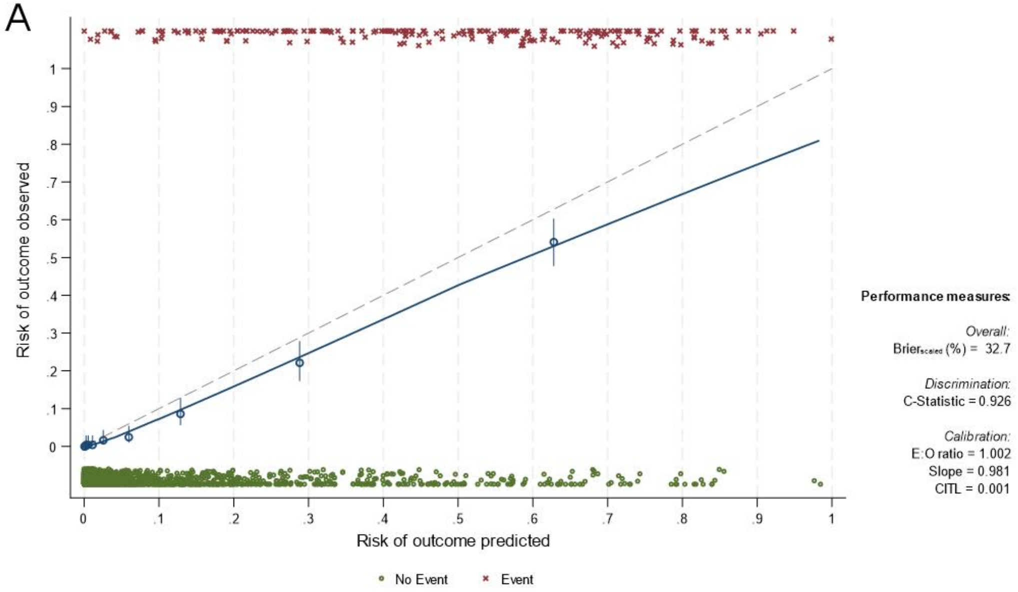

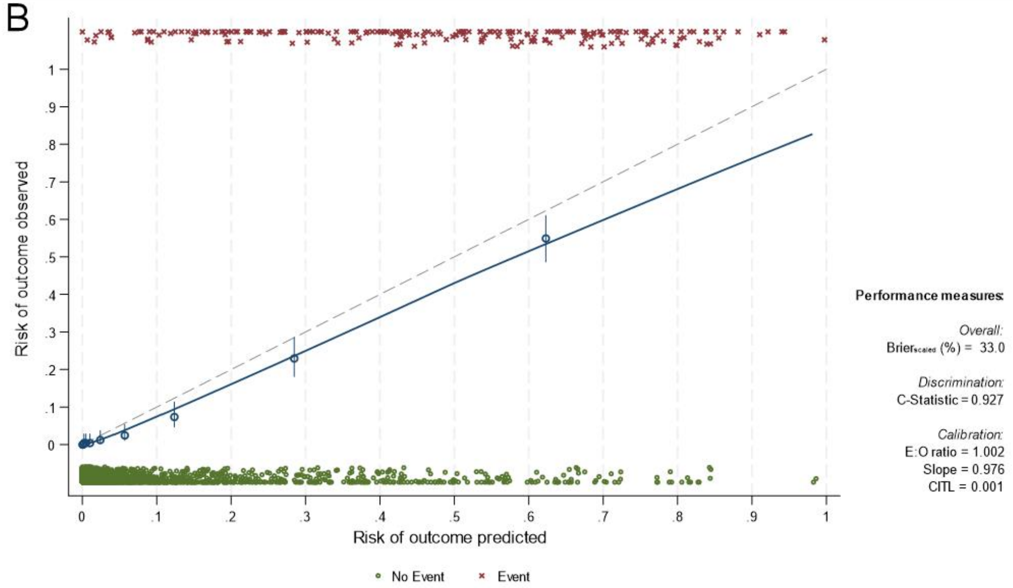

Supplementary Figure 1. Bootstrap method samples 500 times in the derivation cohort for model 1 (A) and model 2 (B).
